# Influence of Vegetation Restoration on Topsoil Organic Carbon in a Small Catchment of the Loess Hilly Region, China

**DOI:** 10.1371/journal.pone.0094489

**Published:** 2014-06-13

**Authors:** Yunbin Qin, Zhongbao Xin, Xinxiao Yu, Yuling Xiao

**Affiliations:** Institute of Soil and Water Conservation, Beijing Forestry University, Beijing, China; University of Vigo, Spain

## Abstract

Understanding effects of land-use changes driven by the implementation of the “Grain for Green” project and the corresponding changes in soil organic carbon (SOC) storage is important in evaluating the environmental benefits of this ecological restoration project. The goals of this study were to quantify the current soil organic carbon density (SOCD) in different land-use types [cultivated land, abandoned land (cessation of farming), woodland, wild grassland and orchards] in a catchment of the loess hilly and gully region of China to evaluate the benefits of SOC sequestration achieved by vegetation restoration in the past 10 years as well as to discuss uncertain factors affecting future SOC sequestration. Based on soil surveys (N = 83) and laboratory analyses, the results show that the topsoil (0–20 cm) SOCD was 20.44 Mg/ha in this catchment. Using the SOCD in cultivated lands (19.08 Mg/ha) as a reference, the SOCD in woodlands and abandoned lands was significantly higher by 33.81% and 8.49%, respectively, whereas in orchards, it was lower by 10.80%. The correlation analysis showed that SOC and total nitrogen (TN) were strongly correlated (*R* ^2^ = 0.98) and that the average C∶N (SOC∶TN) ratio was 9.69. With increasing years since planting, the SOCD in woodlands showed a tendency to increase; however, no obvious difference was observed in orchards. A high positive correlation was found between SOCD and elevation (*R* ^2^ = 0.395), but a low positive correlation was found between slope and SOCD (*R^2^* = 0.170, *P* = 0.127). In the past 10 years of restoration, SOC storage did not increase significantly (2.74% or 3706.46 t) in the catchment where the conversion of cultivated land to orchards was the primary restoration pattern. However, the potential contribution of vegetation restoration to SOC sequestration in the next several decades would be massive if the woodland converted from the cropland is well managed and maintained.

## Introduction

Afforestation and other vegetation restoration techniques have been considered effective practices for the sequestration of carbon (C) to mitigate carbon dioxide (CO_2_) concentrations in the atmosphere [1]–[4]. Soil plays an important role in the global carbon cycle. The soil C pool to a one metre depth has been estimated to sequester approximately three times the amount of carbon sequestered by the atmospheric pool and about four times that in the biotic/vegetation pool [5]. Therefore, a relatively small change in the soil C pool can significantly mitigate or enhance CO_2_ concentrations in the atmosphere [6]–[7].

Land-use change can significantly influence the accumulation and release of SOC. When an ecosystem is disturbed by land-use change, the original equilibrium of the soil carbon pool is broken and a new equilibrium is created. During this process, soil may act as either a source or a sink of carbon depending on the ratio between inflows and outflows [8]. Many studies have reviewed the effect of land-use change on SOC. For instance, deforestation for agricultural purposes is the primary reason for the SOC loss. It was reported that approximately 25% of SOC was lost by conversion of primary forest into cropland [9]. Houghton (1999) estimated that 105 PgC was released into the atmosphere due to the conversion of forests to agricultural lands between 1850 and 1990 [10]. However, afforestation and reforestation on agricultural lands have been cited as effective methods for increasing SOC pool and reducing the atmospheric CO_2_ concentration [3], [11]. Morris et al. (2007) observed that placing agricultural soils in deciduous and conifer forests resulted in soil carbon accumulations of 0.35 and 0.26 MgCha^−1^ yr^−1^, respectively. Based on a meta analysis of 33 recent publications, Laganière et al. (2010) reported that afforestation increased SOC stocks by 26% for croplands. Some studies found that croplands that were converted to abandoned lands or grasslands could also increase the SOC storage [9], [13]–[14]. Therefore, understanding the influence of land-use changes on soil organic carbon is an important step in predicting climatic change and developing potential future CO_2_ mitigation strategies.

To improve the carbon sink status of afforeseation or other vegetation restoration methods on agricultural land, it is necessary to understand the control mechanisms of SOC dynamics to allow more carbon storage in soils [1]. A variety of factors will affect the quantity and quality of SOC after land-use changes. For example, climate variations have a significant effect on SOC, including temperature, precipitation, and potential evapotranspiration changes [11], [15]. Laganière et al. (2010) reported that SOC restoration after afforestation was found to vary with the climate zone, with the temperate maritime zone having a higher SOC increase than others by approximately 17%. In addition, landscape and elevation also have a pronounced effect on SOC change at the catchment scale. Slope is an important topographical factor that affects soil erosion and also has an important influence on the soil nutrient loss of the slope surface [16]. Wang et al. (2012) reported that the SOCD was higher in shady slopes than in sunny slopes, and gentle slopes would generally sequester more SOC than steep slopes. Elevation differences can cause climatic and biological changes in the soil-forming environment and can influence the vertical distribution of SOC [18]. In natural ecosystems, it has been extensively documented that carbon and nitrogen are closely related [4], [19]. Increased nitrogen retention may increase the carbon sequestration potential [1]. In Panama, Batterman et al. (2013) found that symbiotic N_2_ fixation has potentially important implications for the ability of tropical forests to sequester CO_2_ [20]. N_2_-fixing tree species accumulated carbon up to nine times faster per individual than neighbouring non-fixing trees. The soil C/N ratio also has a significant effect on the rate of decomposition of organic compounds by soil microorganisms [21].

Soil erosion, as the most widespread form of soil degradation, has a large impact on the global C cycle, causing a severe depletion of SOC pools in the soil [22]–[24]. The total amount of C released by soil erosion each year has been estimated at approximately 4.0–6.0 Pg/year [23]. China's Loess Plateau, covering approximately 6.2×10^5^ km^2^, has the world's most severe soil erosion because of its unusual geographic landscape, soil and climatic conditions, and long history (over 5000 years) of human activity. Over 60–80% of the land in the Loess Plateau has been affected by soil erosion, with an average annual soil erosion of 2000 to 20000 t km^−2^ yr^−1^ [25]–. Severe soil erosion has resulted in land degradation, which was manifested primarily in the thinning of the soil layer, nutrient loss and fertility reduction, which has directly caused decreases in the local farmers' income and has economically and socially hindered sustainable development [27]–[29]. Unreasonable human activities, such as deforestation and tillage on slopes, have further intensified soil erosion and land degradation in the Loess Plateau [30].

Since the 1950s, the Chinese government has launched many large-scale projects to attempt to control soil erosion and restore vegetation in the Loess Plateau, including large-scale afforestation in the 1970s and comprehensive control of soil erosion on the watershed scale in the 1980s and 1990s. Despite these efforts, there have not been significant increases in ecological benefits, which is largely due to the limitations and influences of bad natural conditions and unreasonable ecological restoration techniques. To control soil erosion and improve the quality of the local environment, “Grain for Green” was initiated by the government in the Loess Plateau in 1999. The government demanded that the agricultural lands with a slope of over 25 degrees be converted to forest, terrace orchards or grassland. To compensate famers for their economic loss, they will be given grain, cash and planting stocks by the government as subsidies and incentives for converting cultivated land back to forest, orchards or grassland. Currently, the Loess Plateau environment appears to be experiencing a recovery following more than 10 years of vegetation restoration [31]–[32].

Over the past decade, large-scale vegetation restoration efforts have brought obvious land-use changes to the Loess Plateau, and also have significantly influence on SOC sequestration. Therefore, understanding the effects of these dynamic changes and the corresponding changes in SOC storage caused by land-use change driven by the implementation of the Grain for Green project is important in evaluating the environmental benefits of this ecological restoration project. Recently, many studies have focused on SOC changes induced by the Grain for Green project in the Loess Plateau. These studies have mainly focused on the SOCD of different land-use types, the SOC pool, the rate of SOC change, the variation in SOC among different land-use conversions and factors which have influenced SOC sequestration in the Loess Plateau after vegetation restoration efforts [32]–[34]. However, for most of the researches, the study time period was shorter than 10 years as vegetation restoration was driven by the Grain for Green project. The study areas have been mostly located at the gully region of the Loess Plateau [2], [17], [35]–[36]. Little is known about the gully area of the Loess hilly region. To gain a complete understanding of the SOC change after restoration in this region, we selected the Luoyugou catchment of the Loess Plateau as the study area, which has the typical geomorphologic characteristics of the gully area of the Loess hilly region.

In this study, we hypothesised that the SOCD varied with land-use types in this catchment, and there was an increase in SOC storage of the total catchment after 10 years of restoration. Therefore, the objectives of this study were to (i) quantify the SOCD of different land-use types differ significantly in the study catchment, (ii) analyze those factors affecting SOCD, (iii) estimate the contribution of land-use conversions on SOC sequestration in the study area, in the past 10 years of restoration, and (iv) discuss the uncertainties in potential SOC sequestration during future ecological restoration efforts in the Loess Plateau.

## Materials and Methods

### Ethics statement

The administration of the Tianshui Experiment Station of Soil and Water Conservation, Yellow River Conservancy Commission and local farmers which are the owners of study lands gave permission for this research at each study site. We confirm that the field studies did not involve endangered or protected species.

### Study area

Tianshui city (104°35′–106°44′E, 34°05′–35°10′N) of Gansu province, China, is located in the western side of the Qinling Mountains, which is the transitional zone between the Qinling Mountains and the Loess Plateau and also belongs to the third sub-region of the Loess hilly region in the middle portion of the Yellow River and the second class tributary of the Wei River. This region has the typical geomorphology of the gully area of the Loess hilly region, which includes earth-rocky mountainous areas, Loess ridges and hilly areas. In this area, the catchment landscape is an agroforestry landscape, and the climate has obvious transitional characteristics. Because of these special geomorphologic characteristics, Walter Lowermilk, deputy director of the US Department of Soil Conservation Service, chose this region in which to build an experimental station for soil and water conservation in 1941. Tianshui station, Suide station and Xifeng station now are three the best-known experimental stations for soil and water conservation in the Loess Plateau.

**Figure 1:**
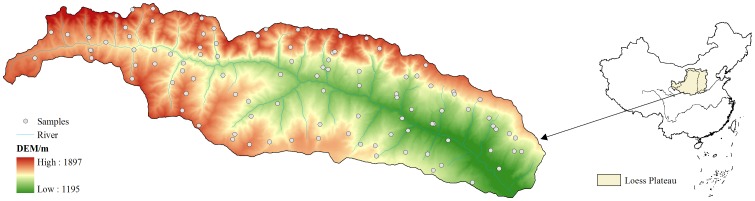
Location of the study area and distribution of the sampling points.

The Luoyugou catchment (105°30′–105°45′E, 34°34′–34°40′N), located in northern Tianshui, is a part of the observation area of the Tianshui soil and water conservation station. Its total area is 72.79 km^2^, with a range from 1165–1895 m above sea level. The main topographic type is loess ridge landform, and the average slope is 19°. It has a typical continental monsoon climate with a mean annual precipitation of 548.9 mm (1986–2004). Approximately 78% of the rainfall is concentrated between July and September. The average annual temperature is 11.4°C (1986–2004),and the annual evaporation is 1293.3 mm. The main soil type in this catchment is mountain grey cinnamon soil, which, according to the Food and Agriculture Organization of the United Nations Educational, Scientific and Cultural Organization (FAO-UNESCO), belongs to the Cambisol soil group and accounts for 91.7% of the soil in the region [37]–[38] (Figure 1).

The main land-use types for the Luoyugou catchment include woodland, wild grassland, orchards, cultivated land, and abandoned land. In the woodland, the major tree species is black locust (*Robinia pseudoacacia L.*), most of which was artificially planted. Woodlands that are more than 30 years old have good water condition and low density because planting is located mainly beside the place beside water ditches and shady slope. They are the retained trees of the planting projects in the late 1950s to 1970s, without human management now. And most of woodland at the age of 10 to 30 years since planting is found in Fenghuang forest farm located in the gully head of this catchment, which has good management and protecting. Since 1999, the government of Tianshui city has widely implemented the “Grain for Green” project. A lot of cultivated land in the ridge top and slope top has been converted into woodland. Most of the woodland is less than 10 years since planting, and has a high density and bad management. Wild grassland is usually found on stone mountain of the northern catchment where the soil layer is thin and slope is steep. Human activities are restricted in there, only sometimes sheep may reach. The major species are dahurian bushclover (*Lespedeza dahurica (Laxm.) Schindl.*), russian wormwood (*Artemsia sacrorum Ledeb.*), digitate goldenbeard (*Bothriochloa ischaemun* (*L.*) *Keng*) and bunge needlegrass (*Stipa bungeana Trin.*), etc. The main fruit species under orchards are cherry (*Cerasus pseudocerasus* (*Lindl.*) *G. Don*), apple (*Malus pumila Mill.*) and apricot trees (*Armeniaca vulgaris Lam.*). The management system of orchards is the conventional tillage which is that the weeds beneath the trees are eliminated with herbicides and dead leaves, dried fruit and twigs are removed by manual blowers. Besides, there are not irrigation facilities and the tree water demand all comes from the rain. Fertiliser and pesticide are also applied in the orchards. This catchment is a rainfed agriculture region with a long cultivated history. The major species in the cultivated land are wheat (*Triticum aestivum L.*), maize (*Zea mays L.*) and edible rape (*Brassica campestris L.*), etc. Abandoned land is the cultivated land has been the ceased farming activities, due to the demand of “Grain for Green” project, the bad environment conditions, the shortage of rural labour and the damage by wild animals, etc. After cultivated land abandoned, old field are spontaneously colonised by various plants, while a secondary succession process will gradually develop during different plant communities. Because of short time since abandoned, the main species in the abandoned land are annual and biennial herb and a bit of perennial herb, such as virgate wormwood (*Artemisia scoparia Waldst. et Kit.*) and green bristlegrass herb (*Utricularia australis R. Br.*).

### Soil sampling and laboratory analysis

Based on the main land-use types and the percentage of each land-use-type area in the total catchment area (2008), we randomly selected 83 land-use blocks that included cultivated land (34 samples), abandoned cropland (9 samples), orchards (13 samples), wild grassland (8 samples) and woodland (19 samples) as investigation plots. Their latitudes and longitudes are shown in Table 1. Spatially separated plots were located at least 3 km from one another to help avoid pseudo-replication. In July 2012, the selected plots were surveyed and collected in the field using the Global Positioning System (GPS). The information recorded from the sampling plots in the field included geographic coordinates, slope, elevation, plant species, and water conservation measures. The years since planting or abandoned of the sampling plots were evaluated by recording tree diameters and heights, plant composition, the degree of decomposition of the topsoil crust and the crop's residual body, and by interviewing local farmers. In each sampling plot — woodland and orchards (10×10 m), cultivated land, abandoned land and wild grass land (5×5 m) — we randomly collected three soil samples using a stainless steel cutting ring 5.0 cm high and 5.0 cm in diameter to measure the soil bulk density (BD) of the topsoil layer (0–20 cm) and by taking five random soil samples between 0 and 20 cm depth with a 20 cm long soil auger. The five soil samples were manually homogenised to form a composite sample for each sampling plot, and a quarter of this sample was taken to the laboratory. These samples were air-dried and passed through a 2 mm sieve, while gravel and roots were removed from each soil sample. A quarter of each sub-sample was completely passed through a 0.25 mm sieve to determine SOC and TN. SOC was determined by the K_2_Cr_2_O_7_-H_2_SO_4_ Walkey-Black oxidation method [39]. TN was measured using the micro-Kjeldahl procedure [40].

**Table 1:** Attributes of the studied sites.

Land-use	Sample sites	Years since planting (abandoned)/a	Elevation/m	Slope/(°)	Bulk density/(g/cm^3^)	Plants
Cultivated land	34	—	1536.94	4.85	1.29	Wheat, maize, rape
Abandoned cropland	9	8–10a	1559.40	4.22	1.35	*Artemisia scoparia*, *Leymus secalinus*
Orchards	13	<5a, 5–10a, >10a	1446.45	1.92	1.32	Cherry, apple, pear
Woodland	24	<10a, 10a–30a, >30a	1639.14	12.13	1.39	Robinia
Wild grassland	8	—	1526.55	13.00	1.44	*Artemisia sacrorum var. incana mattf*, *Stipa bungean*a, *Bothriochloa ischaemun*

### Statistical analysis

The SOCD was calculated using the following equation:
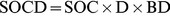
(1)where SOCD was the soil organic carbon density (Mg ha^−1^), SOC was the soil organic carbon content (%), D was the soil layer depth (cm), BD was the soil bulk density (g cm^−3^).

The soil organic carbon storage (SOCS) was calculated using the following equation:
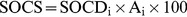
(2)where SOCS was the soil organic carbon storage (t), SOCD_i_ was the soil organic carbon density in the i land-use (Mg ha^−1^), and A_i_ was the area of the i land-use (km^2^).

The increase in SOCS caused by land-use conversion was calculated using the following equation:
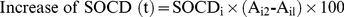
(3)

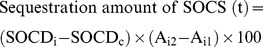
(4)where SOCD_i_ was the SOCD in the i land-use (Mg ha^−1^), A_i1_ and A_i2_ were the i land-use area in 2002 and 2008, respectively (km^2^), and SOCD_c_ was the SOCD of the cultivated land (Mg ha^−1^). All SOCD values of the different land-use types in 2002 and 2008 were the average SOCD values of the soil data collected in 2012.

All data were analysed using Excel 2007 and SPSS (Statistical Package for the Social Sciences) 18.0 software. Analysis of variance (ANOVA) was used to determine the significance of the mean difference. Fisher's LSD test was used to compare the mean values of soil variables when the results of ANOVA were significant at *p*<0.05. Pearson's test was used to analyse the correlation between SOCD and the soil variables. For all analyses, a *p*<0.05 was used to test statistical significance. Finally, SOCD, land-use type, elevation and slope values were used to establish the multiple regression model. SOCD was the dependent variable, and others were the independent variables.

### Satellite data acquisition and processing

In our study, two types of SPOT5 multispectral images were acquired on August 25, 2002 and May 5, 2008, which were multi-spectral images (resolution: 10 m) and panchromatic images (resolution: 2.5 m). To validate the final classification result, we selected 17 points located in the Luoyugou catchment to use as the observed data based on the land-use map and some thematic maps. In July 2012, these monitoring points were collected in the field to validate the panchromatic map, while we recorded 83 points with information of land-use type and longitude and latitude to validate the accuracy of the classification results. Using ArcGIS and ERDAS software, we chose the common pre-classification, which is the supervised classification with a Maximum Likelihood Decision (MLD), to classify the preprocessed images and acquire the data of different land-use types in 2002 and 2008. The overall accuracy and Kappa coefficient of the initial classification images were 87.50% and 0.85 in 2002, respectively, and 90.10% and 0.87 in 2008, respectively. The accuracy of the 88 typical types of land-use information used to validate the classification results was 95.74%, which implied that this classification result could meet the analysis demands.

## Results

### Topsoil organic carbon density under different land-uses

The one-way ANOVA indicated that land-use type had a significant effect on the SOCD (*F* = 8.34, *P*<0.01). However, only the woodland was significantly different from the other land-uses (P<0.05), while no significant differences were found between other land-use types. The average SOCD of the topsoil layer (0–20 cm) in the Luoyugou catchment was 20.44 Mg/ha, and was highest in the woodland and lowest in the orchards (Table 2). Using the SOCD in the cultivated land (19.08 Mg/ha) as a reference, the SOCD in the woodland and abandoned cropland significantly increased by 33.81% and 8.49%, respectively, while the SOCD in the orchards decreased by 10.80%. The SOCD in the wild grassland was close to that of cultivated land. In this area, the coefficient of variation of the SOCD was 26.46%, and was highest (27.08%) in the woodland and smallest (16.75%) in the orchards.

**Table 2:** Topsoil (0–20 cm) SOCD of different land-uses.

Land-use	Total	Woodland	Orchards	Abandoned cropland	Wild grassland	Cultivated land
Sample sites/N	83	19	13	9	8	34
SOCD Mg/ha	20.44	25.53 a	17.02 b	20.70 b	19.35 b	19.08 b
Standard deviation	5.41	6.91	2.85	4.59	3.99	3.66
Minimum	10.47	16.45	11.03	16.55	12.59	10.47
Maximum	40.71	40.71	20.72	31.14	26.02	28.86
Coefficient of variation (%)	26.46	27.08	16.75	22.17	20.62	19.16

* A different letter means a difference significant at 0.05 level.

### Change of topsoil SOC storage in the catchment

Based on the interpretation of the remote sensing data (2000), the results showed that the total area of the four main land-use types including woodland, orchards, cultivated land and wild grassland was 67.5 km^2^, accounted for 92.09% of the total region of the Luoyugou catchment. The difference of 7.02% included river, residential points, and roads that were not taken into account in the calculation of the topsoil SOC storage. From 2002 to 2008, the net decrease in the cultivated land area was 15.11 km^2^, where 7.95 km^2^ of this land was converted into the woodland, and 6.51 km^2^ was converted into the orchards. The increased areas of land were accounted for 52.65% and 43.12% of the total conversion area, respectively. In 2008, the SOC storage in this catchment was 1.39×10^5^ t and was higher than the SOC storage in 2002 (1.35×10^5^ t) by 3706.46 t. When the cultivated land was converted into the woodland, the SOC sequestration contribution was 4484.83 t; however, when the cultivated land was converted into the orchards, the contribution was −723.02 t (Table 3).

**Table 3:** Change in SOC storage from 2002 to 2008 in the Luoyugou catchment.

Land-use	2002			2008			SOC	
	Area/km^2^	SOC		Area/km^2^	SOC			
		Storage/t	Percentage/%		Storage/t	Percentage/%	Increased/t	Sequestration amount/t
Immature woodland	8.16	19764.05	14.62	15.48	37480.99	26.98	17716.94	3759.91
Mature woodland	2.35	7145.31	5.28	2.99	9087.78	6.54	1942.48	724.92
Orchards	4.11	7377.46	5.46	10.62	19082.52	13.74	11705.06	−723.02
Wild grassland	0.71	1374.26	1.02	1.31	2536.62	1.83	1162.36	16.22
Cultivated land	52.18	99557.47	76.63	37.07	70737.09	50.92	−28820.38	–
Total	67.50	135218.55	100.00	67.46	138925.00	100.00	3706.46	3778.03

* Because the area of this catchment in 2008 was less than that in 2002, by 0.04 km^2^, so that about 71.58 t of SOC wasn't taken into the amount of SOC storage. Immature woodland refers to trees under 30 years of age since planting (SOCD = 24.22 Mg/ha), and mature woodland refers to trees more than 30 years of age since planting (SOCD = 30.44 Mg/ha).

### Relationship of soil organic carbon and total nitrogen content

**Figure 2:**
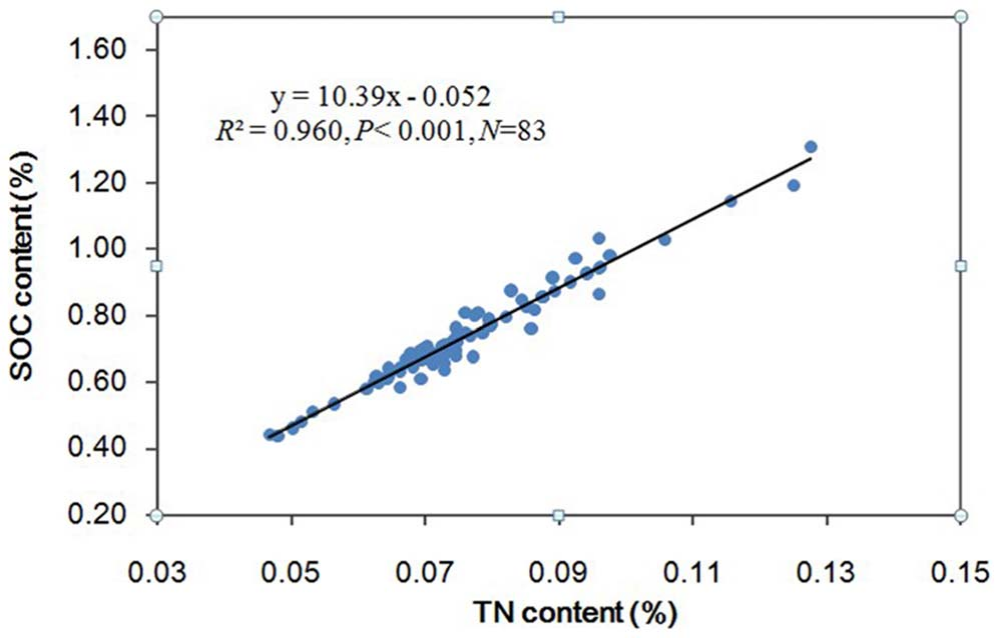
Relationship of topsoil soil organic carbon and total nitrogen content.

In this catchment, soil organic carbon and total nitrogen revealed a significant positive correlation (*R^2^* = 0.978, *p*<0.01) that increased as total nitrogen content and soil organic carbon increased (Figure 2). A one-way ANOVA indicated that land-use had significant effect on the TN (*F* = 3.07. *P* = 0.021), and that had no significant effect on the soil C/N ratio (*F* = 0.86, *P* = 0.48). The average soil C/N was 9.69, with a range of 8.73–10.77. The soil C/N ratio in the woodland was the largest at 9.80 and that in the orchards was the smallest at 9.57. Other land-uses followed the order of abandoned cropland (9.76)>cultivated land (9.66)>wild grassland (9.59).

### Relationship of years since planting and topsoil SOCD

**Figure 3:**
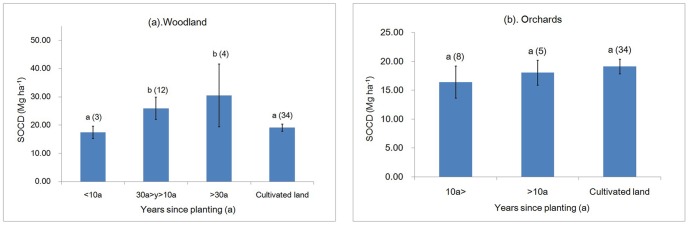
Relationship of years since planting and topsoil SOCD. * The error bars are the standard errors of the mean of SOCD and values above the bars is the number of observations (in parentheses). A different letter means a difference significant at *P*<0.05.

For the woodland, years since planting had a significant effect on SOCD (*F* = 13.00, *P*<0.001). SOCD increased as years since planting increased. However, SOC was lost initially after afforestation in the cultivated land. The SOCD of the woodland that was older than 30 years was higher than that of the woodland under 10 years old (17.45 Mg/ha) and the cultivated land (19.08 Mg/ha), improving by 74.44% and 59.54% respectively (Figure 3a). However, years since planting had no significant effect on SOCD in the orchards (F = 2.01, *P* = 0.146), where the SOCD at >10a was 18.04 Mg/ha and was 10.13% higher at <10a. But, they all were below that of the cultivated land (Figure 3b).

### Relationship of elevation and topsoil SOCD

**Figure 4:**
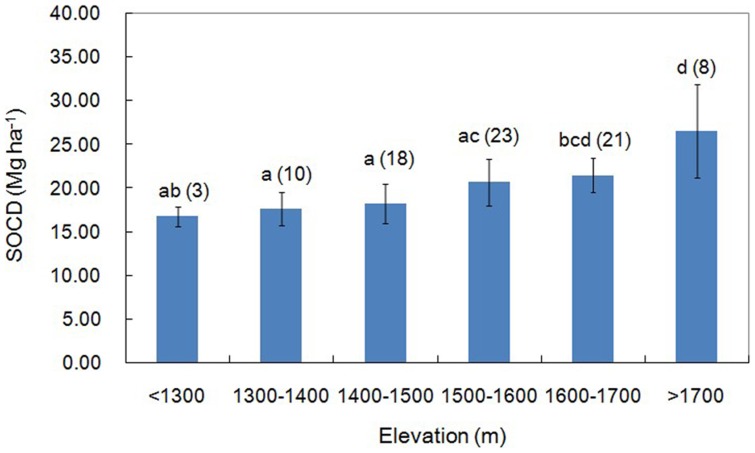
Relationship of elevation and topsoil SOCD. * The error bars are the standard errors of the mean of SOCD and values above the bars is the number of observations (in parentheses). A different letter means a difference significant at *P*<0.05.

The correlation analysis showed that there was a significant positive correlation between elevation and SOCD (*R^2^* = 0.395 *P*<0.01), using the land-use type as the covariate. The SOCD was calculated as the average value with each 100 m used as an elevation gradient. The results showed that with increasing elevation, SOCD had an increasing trend (Figure 4). At an elevation gradient of ≥1700 m, the SOCD was 26.57 Mg/ha, which was higher than that at the elevation gradient of <1300 m by approximately 58.10%.

### Relationship of slope and topsoil SOCD

A one-way ANOVA indicated that land-use had a significant effect on slope (*F* = 2.87, *P* = 0.028) where the average slopes of the woodland and wild grassland all were more than 11.00°, and the average slopes of the cultivated land, abandoned cropland and orchards all were less than 5.00° (Table 4). The correlation analysis showed a low positive correlation between slope and SOCD (*R^2^* = 0.170, *P* = 0.127). A negative correlation was found between the slope and SOCD in the cultivated land, abandoned cropland and orchards (*R* ^2^ = −0.210, *F* = 0.123), and a positive correlation was found in the woodland and wild grassland (*R* ^2^ = 0.250, *F* = 0.209). For all correlations, the land-use type was used as the covariate.

**Table 4:** Slope of different land-uses.

Land-use	Total	Woodland	Orchards	Abandoned cropland	Wild grassland	Cultivated land
Sample sites/N	83	19	13	9	8	32
Slope/°	6.65	11.58 a	1.92 b	4.22 ab	13.00 a	4.85 b
Standard deviation	10.85	14.76	4.80	8.39	12.10	8.93
Minimum	0	0	0	0	0	0
Maximum	45.00	45.00	15.00	20.00	30.00	30.00
Coefficient of variation %	163.16	127.46	250.00	198.82	93.08	184.12
Correlation between slope and SOCD/R^2^	0.170	0.269	−0.169	−0.459	0.480	−0.229

* A different letter means a difference significant at 0.05 level. The signification of correlation between slope and SOCD all were more than 0.05 (*P*>0.05).

### Influence of multivariates on topsoil SOCD

Through normalisation processing of SOCD, elevation and slope and setting land-use dummy variables, the multiple regression model was established using SPSS software. The results were as follows:
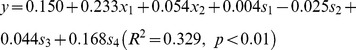
(5)where y was the topsoil SOCD, x_1_ was elevation, x_2_ was slope, s_1_ was wild grassland, s_2_ was orchards, s_3_ was abandoned land, and s_4_ was woodland. The cultivated land was used as the reference when the value of s_1–4_ was 0.

According to the multiple model, elevation, slope and land-use accounted for 32.9% of the SOCD variation using the cultivated land as the reference (Eqn. 5).

## Discussion

### SOC sequestration in different ecological restoration types

The different types of conversion result in different trends in SOC [8]. In this study, the highest SOCD in the woodland implied that the conversion from the cultivated land to the woodland may lead to the accumulation of more SOC than when cultivated land is converted into the orchards or abandoned cropland in this area (Table 2). Some previous research has also found that the conversion from cultivated land to woodland can increase SOC levels [3], [17]. This is mainly because the conversion from cultivated land to woodland can increase topsoil SOCD through increasing biomass inputs into the soil, and reducing soil erosion [9], [12]. In addition, compared to frequent human activity such as tillage and grazing, in the cultivated land and abandoned cropland, less human disturbance in the woodland also enhanced SOC accumulation.

Our research found that the SOCD in the immature woodland (<10 a) was lower than that in the cultivated land. However, the SOCD of the mature woodland (>30 a) was higher than that of the cultivated land, increasing by 59.54% (Figure 3a). Therefore, in the initial conversion from the cultivated land to the woodland, SOCD may decrease, and from 10a after afforestation SOCD increase rapidly and significantly. Other studies have found similar trends [4], [15]. Degryze et al. (2004) in Michigan found no difference in topsoil (0–25 cm) soil C during the first ten years after afforesting in a cropland; however, soil C of the native forest (48.6 t/ha) was significantly greater than the cropland (31.9 t/ha) [41]. Lu et al. (2013), studying afforestation in the Loess Plateau, found that the time of the SOC source to sink transition was 3 to 8 years after afforestation. The decrease of SOC in the first few years following afforestation has been attributed to low net primary productivity of plants, decreased litter inputs and increased decomposition rates [12], [29].

In this study, the topsoil SOCD in the orchards was less than that in the cultivated land, which implied that a decrease in SOC may occur when the cultivated land is converted into the orchards (Figure 3b). This result is similar to the results of Yang et al. in the gully region of the Loess Plateau [42]. However, Xue et al. (2011) reported that when the slope farmland was converted into the orchards, the SOC content increased slowly with increasing years since planting and reached its peak between 20 to 30 years [43]. Thirty years later, the SOC content of the converted orchards was 4.96 g/kg and was higher than that in the slope farmland by 97%. One difference between our study and that by Xue et al. (2011) is the different reference used. The reference in their study was slope cropland, whereas we used the average value of the slope and terrace cropland as the reference. Moreover, management measures in their study were better than ours. For example, they interplanted crops into the fruit trees at the seedling stage and used more fertiliser.

Management measures are one of the main factors affecting the loss of SOC in orchards. In this catchment, fruit, trimmed branches, and litter are removed from orchards, which can cause less organic compounds to enter the soil. Clear tillage in the orchards also causes more soil erosion. Therefore, in some farmland regions that are returning to their historic uses and thus need to develop fruit trees for economic purposes, methods for reducing the loss of SOC and enhancing SOC sequestration have become important. To manage this problem, some researchers have found that orchards managed with conservation practices such as growing grass and leguminous cover crop, mulching the ground, and frequently using organic and inorganic fertilisers could reduce soil erosion and improve the SOC content [44]–[45]. In addition, establishing new orchard management models oriented to SOC sequestration is also very necessary.

Many studies have found that the land-use change from the cultivated land to the abandoned land can increase the SOC stored in soil [13]–[14]. In our study, our results also showed that the SOCD may improve when the cultivated land is converted into the abandoned land (Table 2). With the termination of human disturbance, vegetation in the abandoned land may begin the process of self-succession [14], gradually forming original vegetation communities of their region, which are secondary wild grassland or secondary forest communities. However, this process of succession is slow, as the process that turns the abandoned land into the top secondary bunge needlegrass (*Stipa bungeana Trin.*) takes 40 to 50 years [46]. Further studies are needed to understand the methods needed to accelerate community succession and better sequester SOC in the abandoned land.

The research of Li et al. (2007) in the northern region of the Loess Plateau found that the SOCD of abandoned land at approximately 10 years of age was slightly lower than that in the secondary wild land where planted grassland (*Medicago sativa*) changed in the same number of years. After 6 to 10 years of restoration, degraded artificial grassland may form secondary bunge needlegrass (*Stipa bungeana Trin.*) [47]–[48]. Therefore, planting artificial grass in the abandoned land can shorten the time of succession, which ensures ecological benefits and increase economic benefits at the same time. However, in this study, the SOCD of the abandoned land at 8–10 years of age was higher than in the wild grassland, which was different to the results of previous studies [47]. The main reason for this may be that these grasslands were distributed in the barren stony mountainous area. The average slope of these regions was 13°, and soil erosion was relatively severe. These factors led to low SOCD.

### Benefits of SOC sequestration in the entire region

The SOC storage is determined by the dynamic equilibrium of SOC input and output. After approximately 10 years of ecological restoration, the area of cultivated land greatly decreased, while the area of woodland and orchards increased. From 2002 to 2008, the SOC storage of the entire area only improved by 2.74% (3706.46 t) (Table 3). Therefore, the ecological restoration has an increase in SOC storage, but not significant in the short-term. The main reason for this is that an increase in the SOC resulting from the woodland accumulation was offset by a loss in the SOC resulting from the orchards and the initial conversion stage. So, the SOC storage of the total area is in a relatively stable state. If all of the woodland values are calculated using the SOCD value of the woodland more than 30 years old (30.44 Mg/ha), then considering the loss of SOC by the orchards, the SOC storage of the total area can improve by 7.12% (9625.59 t). Therefore, SOC storage has great potential to increase in the future if the woodland converted from the cropland is well manage and maintain. Although SOC storage has not increased in the past 10 years of restoration, with increasing ecological restoration, soil erosion has been controlled and the quality of the ecological environment, such as the air, water, vegetable coverage has gradually improved in this catchment.

### Influencing factors on the SOC

Results from this study showed a strong correlation between SOC and TN (Table 2), which was consistent with earlier findings [33], [36]. The trend of C∶N ratio varying with the land-use types was similar to that of SOC. The woodland had a higher C∶N ratio than other land-use types due to increased above and below ground biomass. The most prevalent tree in the woodland is the black locust, which has N-fixing capabilities, increased TN can promote tree growth, which results in an increase in SOC; it was found that the SOC increase was greater than the TN increase [33]. In catchments, elevation is a governing variable because of its effect on various environmental factors, such as temperature and precipitation. Our study showed a significant positive correlation between elevation and the SOCD (Figure 4). With rising elevation, topsoil SOC increased. This phenomenon was similar to results of other studies [18], [49], which can be explained by the lower temperatures and increased precipitation with higher elevations. Increased precipitation can promote vegetation growth, which increases the accumulation of humus, and lower temperatures can limit the decomposition and turnover of SOC, leading to enhanced SOC storage.

The slope is one of the main topographical features affecting the soil erosion intensity as well as SOC loss and enrichment [16]. In the present study, land-use types significantly affected the correlation between the slope and SOCD, where there was a positive correlation in woodland and wild grassland and a negative correlation in cultivated land, abandoned land and orchards (Table 4). These outcomes were due mainly to the influence of vegetation type and coverage. Vegetation restoration on slopes such as planting trees and grass can increase surface vegetation coverage and effectively control soil erosion, further reducing the loss of SOC. These results have been found in other regions [17], [30]. Thus, the slope land should be converted to woodland and grassland, or changed to terrace to maintain and increase SOC levels. The multiple regression analysis showed that land-use, slope and elevation accounted for 32.9% of the SOCD variation. The unexplained variation may be caused by the soil particle size, soil aggregates, the aspect and the years of utilisation. Therefore, a benefits evaluation of SOC sequestration achieved through ecological restoration efforts should fully consider those influencing factors.

### Uncertainties over SOC sequestration from future ecological restoration efforts

With ecological restoration efforts increasing, soil erosion in the Loess Plateau has significantly decreased, and ecological benefits such as vegetation coverage have gradually increased. However, in the long run, there are still uncertainties that will persist if we use the present SOC sequestration achievements from ecological restoration to evaluate possible future SOC sequestration benefits.

1. Influence of national policies: with the economy developing, the present subsidy standard for returning farmland to forests in the Loess Plateau has been increasingly unable to meet the basic living demands of local farmers. The competitiveness of this subsidy is also growing increasingly weak due to the influence of factors which are raising grain prices and creating policies which benefit farmers, including the abolition of the agricultural tax and the enhancement of subsidies for farming. Based on the results of 2000 farmers' surveys, Cao et al. (2009) found that approximately 37.2% of farmers planned to return to cultivating forested areas and grasslands once the project's subsidies end in 2018. The smaller the subsidy amount to farmers, the fewer the farmers who are willing to participate in them. If the government is unable to improve farmers' income, much of the vegetation restored during the “Grain for Green” project is at risk of being re-converted into farmland and rangeland at the end of this project [50]. Therefore, it is very important and urgent to determine how to effectively consolidate the achievements of the “Grain for Green” project, especially once the project ends, while continually completing the task of returning farmland to forest. However, the government does not do well in this respect. Therefore, uncertainties exist concerning the benefits from SOC sequestration by ecological restoration in the future.
2. Influence of the environment: Most regions converted from cultivated land to forest are experiencing drought and soil impoverishment. There are no rich water resources in the Loess Plateau, and large-scale vegetation construction will certainly consume massive amounts of water. Lü et al. (2012) found that over half of the Loess Plateau experienced a decrease in runoff (2–37 mm/year) with an average of 10.3 mm/year after the implementation of the “Grain for Green” project [51]. Therefore, with water consumption increases resulting from vegetation, some regions are likely to face a deficit of water resources or even a possible depletion. Drought, decreased rainfall and the excessive depletion of deep soil water by planted vegetation causes the formation of the dried soil layer [52]. The dried soil layer can lead to land degradation and heavily influences vegetation growth, which forms large-scale, low-efficiency production forests [53]–[54]. Once the dried soil layer appears, it is very difficult for recovery to occur. In the process of implementing “Grain for Green” some improper phenomena have occurred, including the improper selection of vegetation restoration types and excessive close planting conducted in the blind pursuit of economic benefits and political achievement [55], and this has accelerated the formation of the dried soil layer. Therefore, in the long run, ecological restoration in the Loess Plateau is likely to face water storage restrictions. Thus, the benefits of SOC sequestration may be uncertain in the future.
3. Influence of private and local government interests: Many regions would prefer that cultivated land be converted into orchards when the cultivated land needs to be converted due to this conversion type may generate a higher land value. However, the SOC sequestration benefits resulting from this type of conversion are lower than in the conversion from cultivated land to woodland, and this conversion type may even cause SOC loss. Over time, farmers may remove old trees in orchards converted from cultivated land, or abandoned land. In addition, according to changes in market demand, farmers may replace other tree species before the original trees have matured. The non-ecological woodland converted from cultivated land also can be harvested under reasonable conditions. However, the SOC sequestration effects of these changes have not been studied. In the future, many woodlands and orchards converted from cultivated land will almost certainly undergo cutting and regeneration. The benefits of SOC sequestration are uncertain in the future unless we can understand the SOC variation under these changes.
4. The influence of urbanization and economic interests: since the reforms and open policies were established, labour exports from rural areas have increased year after year. Lack of labour has caused the abandonment of large areas of rural cultivated lands [56]. Recent research has shown that the SOC content could increase when cultivated land is converted into abandoned land. However, given the increasing speed of abandonment, it is unclear how long this phenomenon can continue. In addition, these labours may choose to go home to engage in farming in the future, affecting by the economic crisis of the original working regions and other reasons such as older age and homesickness. This transform may bring new pressure on the local ecological environment, which may cause the loss of SOC. Therefore, the future benefits of SOC sequestration are uncertain.
5. Influence of climate change: Here, climate change mainly refers to precipitation decline, temperature increase and elevated carbon dioxide levels. According to the results of Xin et al. (2011), the drying trend of the Loess Plateau was highly significant, and annual rainfall showed an obvious decreasing trend over the past five decades (1956–2008) by approximately −1.4 mm/a [57]. The reduced precipitation aggravated the water resource shortage situation in the Loess Plateau, which led to the poor tree growth. This outcome may affect SOC sequestration by vegetation restoration, because the temperature increased and elevated carbon dioxide levels have opposite effects on SOC sequestration. Increasing temperatures may promote SOC decomposition; however, elevated carbon dioxide levels may also improve the net primary productivity of plants and increase both the residual body of vegetation and SOC storage [58]–[59]. Over the last 50 years (1961–2010), the annual mean temperature has significantly increased by 1.91°C in the Loess Plateau [60]. The atmospheric concentration of CO_2_ has increased from 280 p.p.m. to 385 p.p.m. between the pre-industrial era and 2008 [61]–[62]. There is no clear research concerning what functions of the increasing temperatures and elevated carbon dioxide levels for SOC storage are more pronounced. Therefore, some uncertainties exist regarding the possible benefits of SOC sequestration in the future.

Generally speaking, there are many factors that may affect SOC sequestration in the future and may increase or decrease SOC storage. Factors that may be detrimental for SOC storage include: (i) the low national subsidy standard for returning farmland into forest or grasslands and the low income of local farmers; (ii) the persistent, deteriorating environment situation and; (iii) land-use changes caused by private and local government interests. Because of these disadvantageous factors, it is very important to determine how to effectively increase SOC storage. Creating new agriculture products by the implementation of more modern agriculture techniques and offering more work opportunities in urban areas for farmers to increase their income could partially solve these problems. Additionally, the large-scale afforestation that may be exacerbating the soil water shortage in the Loess Plateau should be controlled, especially in vulnerable arid and semi-arid regions, and should fully consider the affordability of such environmental efforts before vegetation restoration is conducted.

## Conclusions

In this study, land-use type had a significant influence on the topsoil SOCD. Compared with the average SOCD of cultivated land, the average SOCD in woodland was significantly larger by 33.81%, while the average SOCD in the orchards decreased by 10.80%. Therefore, the SOCD may improve when cultivated land is converted into woodland and may decline when cultivated land is converted into orchards. Over time, there was an increasing trend of SOCD in woodland, but only a small change of SOCD in the orchards. Based on the remote sensing data on land-use change, we found that the “Grain for Green” project did not significantly increase SOC storage in the Luoyugou catchment in the past 10 years of restoration. This is mainly because the increase of SOC storage by woodland was offset by a SOC storage loss in the orchards and decreases in SOC storage in the initial stage of land-use conversion. If all of the woodland area was calculated as 30.44 t/km^2^ of SOCD, the potential contribution to SOC sequestration in this catchment would increase by 9625.59 t, improving the SOC storage ratio by 7.12%. Therefore, it has a huge potential for future environmental restoration efforts if we could manage and maintain the woodland converted from the cropland well. However, we cannot ignore other factors that affect SOC sequestration, including climate change and national policies.
